# Improving Self-Perceived Emotional Intelligence in Occupational Therapy Students Through Practical Training

**DOI:** 10.3389/fpsyg.2019.00920

**Published:** 2019-04-30

**Authors:** Begoña Polonio-López, José Matías Triviño-Juárez, Ana Isabel Corregidor-Sánchez, Abel Toledano-González, Mª Carmen Rodríguez-Martínez, Pablo Cantero-Garlito, Olga López-Martín, Marta Rodríguez-Hernández, Antonio Segura-Fragoso, Dulce María Romero-Ayuso

**Affiliations:** ^1^Faculty of Health Sciences, University of Castilla-La Mancha, Talavera de la Reina, Spain; ^2^Primary Care Center Zaidín Sur, Andalusian Health Service, Granada, Spain; ^3^Department of Physical Therapy, Occupational Therapy Division, Faculty of Health Sciences, University of Málaga, Málaga, Spain; ^4^Department of Physical Therapy, Occupational Therapy Division, Faculty of Health Sciences, University of Granada, Granada, Spain

**Keywords:** Occupational Therapy, emotional intelligence (EI), practical abilities, students-health occupations, healthcare profession education

## Abstract

**Background:** In the field of healthcare, higher skills in emotional intelligence (EI) have been proven to have a positive impact on healthcare professionals in general and occupational therapists in particular and also on teamwork and patient care. The purpose of this research was to determine whether performing practical work included in the undergraduate Occupational Therapy program improves students' self-perceived EI, and whether there are any differences in the latter depending on the area in which this practical training is performed.

**Methods:** It was conducted a multicenter, quasi-experimental, pre-post study with Occupational Therapy undergraduate students in the 2016–2017 academic year. A total of 184 students met the inclusion criteria. Of these, 10 (5.40%) declined to participate; therefore, the participation rate was 94.60% and the initial sample comprised 174 students. Mean age was 21.34 years (*SD* = 2.54) and 84.50% (*n* = 147) were women. Students' self-perceived EI was measured with the Trait Meta-Mood Scale-24 (TMMS-24). This measure was completed in the two weeks prior to the start of the practical training period and one week after the end of it. McNemar-Bowker and McNemar tests were used to analyze the differences in self-perceived EI between these two points in time.

**Results:** After the practical training, an improvement in self-perceived EI was observed in women (Emotional Attention, Emotional Clarity, and Emotional Regulation dimensions). Regarding areas of practical training, results showed an improvement in EI in women who had received practical training in the areas of Physical Rehabilitation, Mental Health and Geriatrics and Gerontology.

**Conclusions:** The findings contribute to a better understanding of the relation between practical training and an improvement in self-perceived EI. This insight can help make changes in the teaching methodology to enhance the emotional skills needed for a better professional performance.

## Introduction

Emotional intelligence (EI) was first defined by Salovey and Mayer as “the ability to monitor one's own and others' feelings, to discriminate among them and to use this information to guide one's thinking and action” (pp. 189) (Salovey and Mayer, 1990). EI has been understood mainly from two perspectives. The first one is that of a set of abilities for emotional processing, according to a skill model (Salovey and Mayer, 1990; Mayer and Salovey, 1997; Mayer et al., 2011). These authors propose a model of four separate abilities or “four-branch model,” on which the present study is based: (a) emotional perception, understood as the ability to perceive emotions in oneself and others; (b) emotional facilitation, that is, the ability to generate, use and feel emotions necessary to communicate feelings or use them in cognitive processes; (c) emotional understanding, which refers to the ability to understand emotional information, how emotions combine and progress through interpersonal relationships and the ability to reason about emotional meanings; and (d) emotional regulation, that is, the ability to open oneself to feelings, to modulate them and regulate them in oneself (Mayer and Salovey, 1997). In this model, EI is understood as a skill that can improve over time. The second perspective is that of a personality trait, according to mixed perspective models (Goleman, 1995; Cooper and Sawaf, 1997; Bar-On, 2006), in which it propose that EI comprises emotion-related personality traits (e.g., optimism) in addition to socio-emotional skills and motivational aspects (Petrides and Furnham, 2000).

EI is essential in healthcare professionals in general and occupational therapists in particular. It promotes the development of self-regulatory mechanisms that reduce individuals' readiness to respond automatically to intrinsic and extrinsic stimuli; it increases the tendency to set targets and goals that lead to a regulation of responses to environmental stimuli at the emotional, cognitive and behavioral levels (Moreira et al., 2014). When EI is combined with appropriate knowledge, clinical reasoning skills, professional behavior, and ethical values, students are able to become competent professionals (Gribble et al., 2017).

In Occupational Therapy (OT), practical training of the students is essential in addition to theoretical teaching and can modify their EI (Gribble et al., 2018). According to the guidelines of the World Federation of Occupational Therapists (WFOT), in the undergraduate OT program, practical training must include a minimum of 1,000 h of direct clinical practice (World Federation of Occupational Therapists, 2016). Contact of students with experienced practitioners and patients gives them the opportunity to relate their theoretical knowledge to real-life practice, socialize with their future profession, boost their confidence regarding patient care and develop their professional identity (Goldie, 2012; Arreciado Marañón and Isla Pera, 2015; McCloughen and Foster, 2017).

There are studies that have explored the relation between EI and a better achievement of OT students in practical training. One study showed a positive correlation between EI and the performance of OT students in academic training (Andonian, 2013). Other authors found that EI was a significant predictor of work performance, highlighting its importance in practical training (Brown et al., 2016). Another study revealed that EI—specifically emotional reasoning, the management of others' emotions, extroversion, and emotional stability in OT students—was a predictor of teamwork skills (Brown et al., 2017). However, research focused on changes in EI of OT students after their practical training are few and show inconclusive results. A longitudinal study conducted with students of OT, Speech Therapy and Physiotherapy, showed that the EI dimensions studied did not improve after practical training (Gribble et al., 2017). By contrast, another longitudinal study found a significant improvement in the overall EI score of OT students after practical training, as well as an increase in self-perception, decision making, self-realization, emotional self-awareness, independence, and objectivity scores (Gribble et al., 2018). It is worth noting that none of the aforementioned studies took into account the specific areas in which the OT students undertook their practical training, a factor that may be related to changes in self-perceived EI.

Considering this, our objectives were to determine if there were any changes in the self-perceived EI of OT students after exposure to their first practical training rotation, and if changes in EI were related to the area of clinical practice. Thus, the following hypotheses were formulated:

Hypothesis 1: Self-perceived EI will improve after practical training in OT undergraduate students.
Hypothesis 2: Changes in self-perceived EI will be related to the area of practical training.

## Materials and Methods

### Design

Multi-center, quasi-experimental, pre-post study.

#### Participants and Procedure

Participants were OT undergraduate students at the universities of Castilla-La Mancha (UCLM) and Malaga (UMA). Both are public universities in Spain. The recruitment process took place in the 2016–2017 academic year. Participation was voluntary and free, and candidates were selected using non-probabilistic convenience sampling. Inclusion criteria were not having received any practical training and having given written informed consent to take part in the study. At UCLM, two different curricula coexisted in 2016–2017: in one of them, which was being phased out, students began their practical training in the third year (OT 311 Degree Plan); in the other, which was new, practical training started in the second year (OT 382 Degree Plan). At UMA, only one curriculum was in force in the 2016-2017 academic year, and students started their practical training in the third year. At both UCLM and UMA, the first practical rotation takes place in the second semester of the academic year and lasts between 4 and 6 weeks.

**Figure 1:**
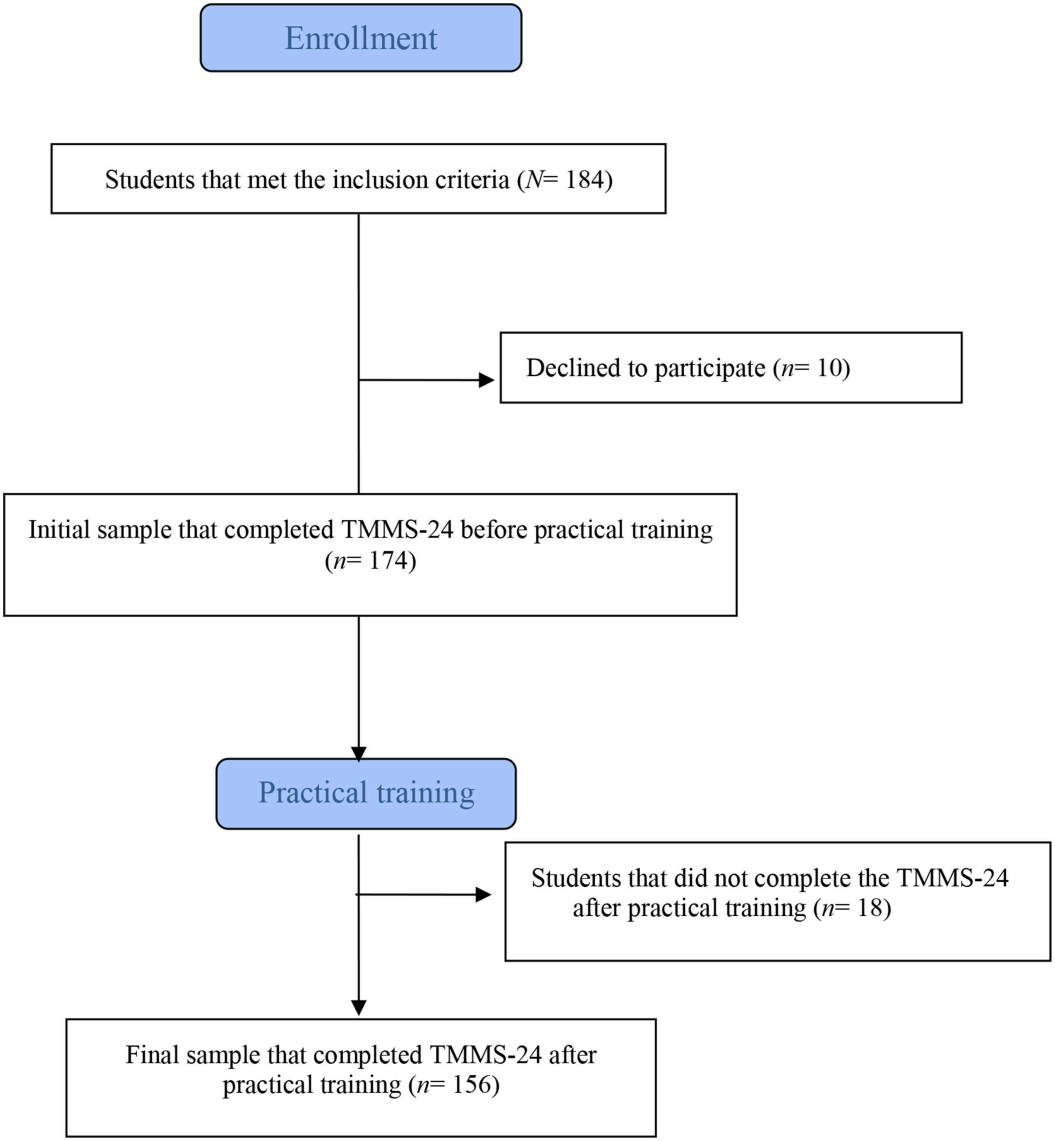
Diagram of subject recruitment.

A total of 184 students met the inclusion criteria. Of these, 10 (5.40%) declined to be included in the study; therefore, the participation rate was 94.60% and the initial sample comprised 174 OT undergraduate students (Figure 1). Mean age was 21.34 years (*SD* = 2.54), 96.60% of students (*n* = 168) were Spanish, 84.50% (*n* = 147) were women, 60.30% (*n* = 105) were students at UCLM, and 31% (*n* = 54) were in second year (Table 1). The 174 students completed the required questionnaires (i.e., sociodemographic variables and Trait Meta-Mood Scale-24 [TMMS-24]) 2 weeks prior to the start of the practical training period. Of these, 156 (89.65%) completed the TMMS-24 1 week after the end of their practical training, and also indicated the area in which they had performed it. No significant differences were found in sociodemographic variables (i.e., age, nationality, sex, university of origin, year of study) between the sample of students who completed the TMMS-24 both before and after the practical training (*n* = 156) and those who only completed it before the practical training (*n* = 18). Regarding the areas of practical training, 8% of male students (*n* = 2) underwent it in Child Care, 20% (*n* = 5) in Physical Rehabilitation, 12% (*n* = 3) in Mental Health, 56% (*n* = 14) in Geriatrics and Gerontology, and 4% (*n* = 1) in other non-conventional areas. In the case of female students, 4.6% (*n* = 6) performed their practical training in Child Care, 31.30% (*n* = 41) in Physical Rehabilitation, 21.40% (*n* = 28) in Mental Health, 37.40% (*n* = 49) in Geriatrics and Gerontology, and 5.3% (*n* = 7) in other non-conventional areas.

**Table 1:** Sociodemographic characteristics (*N* = 174).

		**Mean**	***SD***
Age		21.34	2.54
		***n***	**%**
**NATIONALITY**			
	Spanish	168	96.60
	Other	6	3.40
**SEX**			
	Female	147	84.50
	Male	27	15.50
**UNIVERSITY OF ORIGIN**			
	UCLM	105	60.30
	UMA	69	39.70
**YEAR**			
	Second	54	31
	Third	120	69

*UCLM, University of Castilla-La Mancha; UMA, University of Malaga*.

#### Data Collection and Instruments

In the 2 weeks prior to the start of the practical training period, participants completed a questionnaire that included sociodemographic data (i.e., nationality, age, sex, university, and year of study). Along with this questionnaire, students completed the TMMS-24, a self-reported measure based on the Trait Meta-Mood Scale (TMMS-48) developed by the research group of Salovey, Mayer, Goldman, Turvey, and Palfai (Salovey et al., 1995) to determine the self-perceived EI. This measure was completed again by the same students 1 week after the end of the practical training period. The TMMS-24 is a shorter version of the scale that was adapted to the Spanish population (Fernández-Berrocal et al., 1998; Fernandez-Berrocal et al., 2004). This scale comprises the following three key dimensions of EI: 1. Emotional Attention (EA), defined as the ability to perceive and express feelings appropriately; 2. Emotional Clarity (EC), which corresponds to the understanding of emotional states; and 3. Emotional Repair (ER), which refers to the ability to regulate emotional states correctly, blocking negative moods and prolonging positive moods. Each dimension consists of 8 items, scored on a 5-point Likert scale ranging from 1 (strongly agree) to 5 (strongly disagree). Depending on the responses obtained, the total score for each of the dimensions is ranged from 8 to 40 points and divided into three categories. In EA, a score of ≤21 for men (≤24 for women) corresponds to the category “Must improve attention: pays insufficient attention”; a score of 22-32 points (25-35 points for women) corresponds to “Adequate attention”; and a score of ≥33 (≥36 for women) indicates “Must improve attention: pays too much attention.” Regarding EC, a score of ≤25 for men (≤23 for women) corresponds to the category “Must improve clarity”; a score of 26-35 points (24-34 points for women) refers to “Adequate clarity”; and a score of ≥36 (≥35 for women) indicates “Excellent clarity.” In ER, a score of ≤23 in both men and women indicates “Must improve repair”; a score of 24-35 points (24-34 points for women) refers to “Adequate repair”; and a score of ≥36 (≥35 for women) corresponds to “Excellent repair.” The TMMS-24 has shown good internal consistency for the dimensions EA (α = 0.90), EC (α = 0.90), and ER (α = 0.86), and adequate test-retest reliability for such dimensions, with values of 0.60, 0.70, and 0.83, respectively (Fernandez-Berrocal et al., 2004). In this study, the internal consistency of the TMMS-24 before and after practical training was good, with a Cronbach α of 0.89 and 0.88 for EA dimension, 0.81 and 0.90 for EC dimension, and 0.82 and 0.86 for ER dimension, respectively. Additionally, once the practical training was completed, participants were asked about the specific area in which they had undergone their training: Child Care; Physical Rehabilitation; Mental Health; Geriatrics and Gerontology; other non-conventional areas (e.g., social exclusion, gender violence, prisons).

#### Statistical Analysis

Descriptive statistics for qualitative and quantitative variables were used. Differences in sociodemographic variables between the group of students who completed the study and those who did not were analyzed by means of the *t*-test and Pearson's chi-square test or Fisher's exact test when the expected values in any of the cells of the contingency tables were lower than five (Martínez-González et al., 2009). In order to evaluate the changes in students' self-perceived EI after their exposure to practical training, differences in the three dimensions of EI (EA, EC, and ER) were analyzed with the McNemar-Bowker test. Where differences were significant, the McNemar test was applied to analyze the changes in each dimension, first, without considering the area of practical training, and second, taking this factor into account, and in both cases using the Bonferroni correction for multiple comparisons. EA was considered to improve when the proportion of students in the “Adequate attention” category significantly increased after practical training. EC and ER were both considered to improve if, after the practical training, there was a significant increase in the proportion of students in the “Adequate clarity” and/or “Excellent clarity,” and “Adequate repair” and/or “Excellent repair” categories, respectively. Furthermore, the 95% confidence intervals (95% CI) of the differences of proportions were calculated using the Newcombe's calculation method for differences of proportions of paired populations (Newcombe and Altman, 2000). In the analysis of the changes in each dimension of EI with McNemar test, adjusted using the Bonferroni *post-hoc* correction, the level of statistical significance was set at *p* < 0.017. In the remaining statistical analyses (Pearson's chi-square test, Fisher's exact test, McNemar-Bowker test), the level of statistical significance was set at *p* < 0.05. The statistical analysis was conducted using IBM SPSS software, version 20.

#### Ethics

All candidates gave written informed consent to participate in this project. The study was approved by the Ethics Committee for Scientific Research of the Healthcare Area of Talavera de la Reina (CEIC Code: 6/2017) and by the Ethics and Research Committee of the University of Malaga (CEUMA: 86-2016-H).

## Results

### Changes in EA After the First Period of Practical Training

Men showed significant changes in EA after the practical training (*p* = 0.038) (Table 2). A subsequent analysis of differences using Bonferroni correction didn't show significant differences in EA, either independently of the area of practical training (Table 3) or taking this factor into account. Regarding women, results showed a significant change in EA after the practical training (*p* < 0.001) (Table 2). The analysis of the differences adjusted by Bonferroni correction, found that after the practical training, the proportion of women with adequate EA increased by 41% (*n* = 50) (*p* < 0.001; 95% CI: 31.24–49.34). According to the area, a significant increase in the proportion of women with adequate EA was found in those who had undergone training in the Physical Rehabilitation area (53.85%; *n* = 21) (*p* < 0.001; 95% CI: 34.93–67.04) (Table 4). Similar results were found in those who received their practical training in the area of Mental Health (29.17%; *n* = 7) (*p* = 0.016; 95% CI: 8.44–45.71) and in those who received it in Geriatrics and Gerontology (34.04%; *n* = 16) (*p* < 0.001; 95% CI: 17.75–47.52) (Table 4). However, no significant changes were observed in women who had received practical training in Child Care or in non-conventional areas.

**Table 2:** Self-perceived Emotional Intelligence (TMMS-24) in Occupational Therapy students before and after practical training (*N* = 156).

	**EA after practical training**				
**EA before practical training**	**Must improve EA: pays insufficient attention** ***n* (%)**	**Adequate EA** ***n* (%)**	**Must improve EA: pays excessive attention** ***n* (%)**	**Total** ***n* (%)**	*p-*value^*^
**MEN (*****N*** **=** **25)**					
Must improve EA: pays insufficient attention	3 (12)	8 (32)	2 (8)	13 (52)	0.038
Adequate EA	1 (4)	6 (24)	3 (12)	10 (40)	
Must improve EA: pays excessive attention	0 (0)	1 (4)	1 (4)	2 (8)	
Total *n* (%)	4 (16)	15 (60)	6 (24)	25 (100)	
**WOMEN (*****N*** **=** **131)**					
Must improve EA: pays insufficient attention	40 (30.55)	51 (38.95)	0 (0)	91 (69.50)	<0.001
Adequate EA	1 (0.75)	30 (22.95)	4 (3.00)	35 (26.70)	
Must improve EA: pays excessive attention	0 (0)	2 (1.50)	3 (2.30)	5 (3.80)	
Total *n* (%)	41 (31.30)	83 (63.40)	7 (5.30)	131 (100)	
	**EC after practical training**				
**EC before practical training**	**Must improve EC** ***n*** **(%)**	**Adequate EC** ***n*** **(%)**	**Excellent EC** ***n*** **(%)**	**Total** ***n*** **(%)**	***p-***value^*^
**MEN (*****N*** **=** **25)**					
Must improve EC	10 (40)	9 (36)	0 (0)	19 (76)	0.025
Adequate EC	1 (4)	2 (8)	0 (0)	3 (12)	
Excellent EC	1 (4)	0 (0)	2 (8)	3 (12)	
Total *n* (%)	12 (48)	11 (44)	2 (8)	25 (100)	
**WOMEN (*****N*****=131)**					
Must improve EC	30 (22.95)	56 (42.75)	2 (1.50)	88 (67.20)	<0.001
Adequate EC	0 (0)	23 (17.50)	15 (11.50)	38 (29)	
Excellent EC	0 (0)	3 (2.30)	2 (1.50)	5 (3.80)	
Total *n* (%)	30 (22.95)	82 (62.55)	19 (14.50)	131 (100)	
	**ER after practical training**				
**ER before practical training**	**Must improve ER** ***n*** **(%)**	**Adequate ER** ***n*** **(%)**	**Excellent ER** ***n*** **(%)**	**Total** ***n*** **(%)**	***p-***value^*^
**MEN (*****N*** **=** **25)**					
Must improve ER	5 (20)	5 (20)	1 (4)	11 (44)	0.261
Adequate ER	1 (4)	10 (40)	2 (8)	13 (52)	
Excellent ER	0 (0)	1 (4)	0 (0)	1 (4)	
Total *n* (%)	6 (24)	16 (64)	3 (12)	25 (100)	
**WOMEN (*****N*** **=** **131)**					
Must improve ER	17 (12.95)	50 (38.20)	1 (0.75)	68 (51.90)	<0.001
Adequate ER	6 (4.60)	34 (25.95)	17 (12.95)	57 (43.50)	
Excellent ER	0 (0)	0 (0)	6 (4.60)	6 (4.60)	
Total *n* (%)	23 (17.55)	84 (64.15)	24 (18.30)	131 (100)	

TMMS-24, Trait Meta-Mood Scale-24 emotional intelligence self-report scale; EA, Emotional Attention; EC, Emotional Clarity; ER, Emotional Repair;

* *McNemar-Bowker's test p-value (level of statistical significance set at p < 0.05)*.

**Table 3:** Self-perceived Emotional Intelligence (TMMS-24) in Occupational Therapy students before and after practical training (analysis of the changes using Bonferroni *post-hoc* correction).

	**Pre**		**Post**						**Pre**		**Post**				
**EA MEN**	***n***	**%**	***n***	**%**	**DP (%)**	*p*-value^*^	**CI 95%**	**EA WOMEN**	***n***	**%**	***n***	**%**	**DP (%)**	*p*-value^*^	**CI 95%**
Must improve EA: pays insufficient attention	11	61.10	4	22.20	38.90	0.039	6.97 to 61.53	Must improve EA: pays insufficient attention	91	74.60	41	33.60	41	<0.001	31.24 to 49.34
Adequate EA	7	38.90	14	77.80				Adequate EA	31	25.40	81	66.40			
Must improve EA: pays insufficient attention	5	83.30	3	50	33.33	0.500	−17.17 to 67.43	Must improve EA: pays insufficient attention	40	93	40	93	0	1	−8.30 to 8.30
Must improve EA: pays excessive attention	1	16.70	3	50				Must improve EA: pays excessive attention	3	7	3	7			
Must improve EA: pays excessive attention	2	18.20	4	36.40	18.18	0.625	−18.16 to 49.30	Must improve EA: pays excessive attention	34	87.20	32	82.10	5.13	0.687	−8.78 to 19.30
Adequate EA	9	81.80	7	63.60				Adequate EA	5	12.80	7	17.90			
**EC MEN**	***n***	**%**	***n***	**%**	**DP (%)**	***p***-value^*^	**CI 95%**	**EC WOMEN**	***n***	**%**	***n***	**%**	**DP (%)**	***p***-value^*^	**CI 95%**
Must improve EC	19	86.40	11	50	36.36	0.021	8.80 to 57.60	Must improve EC	86	78.90	30	27.50	51.38	<0.001	40.90 to 59.80
Adequate EC	3	13.60	11	50				Adequate EC	23	21.10	79	72.50			
Must improve EC	10	76.90	11	84.60	7.69	1	−15.17 to 31.04	Must improve EC	32	94.10	30	88.20	5.88	0.5	−5.65 to 19.18
Excellent EC	3	23.10	2	15.40				Excellent EC	2	5.90	4	11.80			
Adequate EC	2	50	2	50	0	1	−35 to 35	Adequate EC	38	88.40	26	60.50	27.90	0.008	9.51 to 44.17
Excellent EC	2	50	2	50				Excellent EC	5	11.60	17	39.50			
**ER MEN**	***n***	**%**	***n***	**%**	**DP (%)**	***p***-value^*^	**CI 95%**	**ER WOMEN**	***n***	**%**	***n***	**%**	**DP (%)**	***p***-value^*^	**CI 95%**
Must improve ER	10	47.60	6	28.60	19.05	0.219	−4.25 to 39.38	Must improve ER	67	62.60	23	21.50	41.12	< 0.001	28.92 to 51.49
Adequate ER	11	52.40	15	71.40				Adequate ER	40	37.40	84	78.50			
Must improve ER	6	100	5	83.30	16.67	NA	NA	Must improve ER	18	75	17	70.80	4.17	1	−8.03 to 16.66
Excellent ER	0	0	1	16.70				Excellent ER	6	25	7	29.20			
Adequate ER	12	92.30	11	84.60	7.69	1	−21.44 to 36.02	Adequate ER	51	89.50	34	59.60	29.80	<0.001	17.15 to 41.95
Excellent ER	1	7.70	2	15.40				Excellent ER	6	10.50	23	40.40			

TMMS-24, Trait Meta-Mood Scale-24 emotional intelligence self-report scale; DP, Difference between correlated proportions or percentages; EA, Emotional Attention; EC, Emotional Clarity; ER, Emotional Repair;

* *McNemar's test p-value, adjusted by Bonferroni post-hoc correction (level of statistical significance set at p < 0.017); NA, Not applicable; CI 95% (95% confidence interval)*.

**Table 4:** Self-perceived Emotional Intelligence (TMMS-24) in Occupational Therapy students (women) before and after practical training, by area of practical work where significant differences were found (analysis of the changes using Bonferroni *post-hoc* correction).

**Physical rehabilitation**	**Pre**	**Post**			
	***n* (%)**	***n* (%)**	**DP (%)**	*p*-value^*^	**CI 95%**
**EA**					
Must improve EA: pays insufficient attention	33 (84.60)	12 (30.80)	53.85	<0.001	34.93 to 67.04
Adequate EA	6 (15.40)	27 (69.20)			
Must improve EA: pays insufficient attention	12 (100)	12 (100)	0	NA	NA
Must improve EA: pays excessive attention	0 (0)	0 (0)			
Adequate EA	8 (100)	6 (75)	25	NA	NA
Must improve EA: pays excessive attention	0 (0)	2 (25)			
**EC**					
Must improve EC	24 (68.60)	8 (22.90)	45.71	<0.001	26.29 to 59.86
Adequate EC	11 (31.40)	27 (77.10)			
Must improve EC	9 (100)	8 (88.90)	11.10	NA	NA
Excellent EC	0 (0)	1 (11.10)			
Adequate EC	16 (100)	11 (68.70)	31.30	NA	NA
Excellent EC	0 (0)	5 (31.30)			
**ER**					
Must improve ER	24 (72.70)	8 (24.20)	48.50	<0.001	25.96 to 64.27
Adequate ER	9 (27.30)	25 (75.80)			
Must improve ER	7 (77.80)	7 (77.80)	0	1	−24.67 to 24.67
Excellent ER	2 (22.20)	2 (22.20)			
Adequate ER	14 (87.50)	8 (50)	37.50	<0.001	8.49 to 59.64
Excellent ER	2 (12.50)	8 (50)			
**Mental health**	**Pre**	**Post**			
	***n*** **(%)**	***n*** **(%)**	**DP (%)**	***p***-value^*^	**CI 95%**
**EA**					
Must improve EA: pays insufficient attention	15 (62.50)	8 (33.30)	29.17	0.016	8.44 to 45.71
Adequate EA	9 (37.50)	16 (66.70)			
Must improve EA: pays insufficient attention	8 (88.90)	8 (88.90)	0	1	−29.56 to 29.56
Must improve EA: pays excessive attention	1 (11.10)	1 (11.10)			
Adequate EA	10 (76.90)	11 (84.60)	7.69	1	−22.76 to 36.84
Must improve EA: pays excessive attention	3 (23.10)	2 (15.40)			
**EC**					
Must improve EC	20 (83.30)	6 (25)	58.30	<0.001	32.54 to 73.67
Adequate EC	4 (16.70)	18 (75)			
Must improve EC	7 (87.50)	6 (75)	12.50	1	−22.79 to 45.87
Excellent EC	1 (12.50)	2 (25)			
Adequate EC	6 (85.70)	4 (57.10)	28.60	0.5	−15.97 to 62.09
Excellent EC	1 (14.30)	3 (42.90)			
**ER**					
Must improve ER	14 (66.70)	6 (28.60)	38.10	0.021	9.65 to 58.71
Adequate ER	7 (33.30)	15 (71.40)			
Must improve ER	6 (85.70)	5 (71.40)	14.30	1	−24.36 to 49.48
Excellent ER	1 (14.30)	2 (28.60)			
Adequate ER	11 (91.70)	6 (50)	41.70	0.063	05.09 to 67.22
Excellent ER	1 (8.30)	6 (50)			
**Geriatrics and gerontology**	**Pre**	**Post**			
	***n*** **(%)**	***n*** **(%)**	**DP (%)**	***p***-value^*^	**CI 95%**
**EA**					
Must improve EA: pays insufficient attention	35 (74.50)	19 (40.40)	34.04	<0.001	17.75 to 47.52
Adequate EA	12 (25.50)	28 (59.60)			
Must improve EA: pays insufficient attention	18 (94.70)	18 (94.70)	0	1	−17.75 to 17.75
Must improve EA: pays excessive attention	1 (5.30)	1 (5.30)			
Adequate EA	12 (92.30)	11 (84.60)	7.69	1	−17.24 to 33.52
Must improve EA: pays excessive attention	1 (7.70)	2 (15.40)			
**EC**					
Must improve EC	34 (82.90)	13 (31.70)	51.22	<0.001	33.03 to 64.29
Adequate EC	7 (17.10)	28 (68.30)			
Must improve EC	13 (100)	13 (100)	0	NA	NA
Excellent EC	0 (0)	0 (0)			
Adequate EC	12 (80)	10 (66.70)	13.33	0.727	−22.45 to 45.25
Excellent EC	3 (20)	5 (33.30)			
**ER**					
Must improve ER	24 (58.50)	7 (17.10)	41.46	<0.001	20.56 to 57.71
Adequate ER	17 (41.50)	34 (82.90)			
Must improve ER	4 (66.70)	4 (66.70)	0	1	−28.63 to 28.63
Excellent ER	2 (33.30)	2 (33.30)			
Adequate ER	20 (90.90)	14 (63.60)	27.27	0.031	5.62 to 47.32
Excellent ER	2 (9.10)	8 (36.40)			

TMMS-24, Trait Meta-Mood Scale-24 emotional intelligence self-report scale; DP, Difference between correlated proportions or percentages; EA, Emotional Attention; EC, Emotional Clarity; ER, Emotional Repair;

* *McNemar's test p-value, adjusted by Bonferroni post-hoc correction (level of statistical significance set at p < 0.017); NA, Not applicable; CI 95% (95% confidence interval)*.

### Changes in EC After the First Period of Practical Training

Men showed significant changes in EC after the period of practical training (*p* = 0.025) (Table 2). The analysis of the differences adjusted by Bonferroni correction didn't found significant differences in EC, either independently of the area of practical training (Table 3) or by area of practical work. In women, results showed a significant change in EC after practical training (*p* < 0.001) (Table 2). A subsequent analysis of differences using Bonferroni correction showed an increase in the proportion of women with adequate EC (51.38%; *n* = 56) (*p* < 0.001; 95% CI: 40.90–59.80) and excellent EC (27.90%; *n* = 12) (*p* = 0.008; 95% CI: 9.51–44.17) after practical training. Regarding the area, a significant increase in the proportion of women with adequate EC was found in those who had undergone practical training in the Physical Rehabilitation area (45.71%; *n* = 16) (*p* < 0.001; 95% CI: 26.29–59.86), in those who had received practical training in Mental Health (58.30%; *n* = 14) (*p* < 0.001; 95% CI: 32.54–73.67), and in those who done so in Geriatrics and Gerontology (51.22%; *n* = 21) (*p* < 0.001; 95% CI: 33.03–64.29) (Table 4). No significant changes were observed in the women who had rotated in the areas of Child Care or in non-conventional areas.

### Changes in ER After the First Period of Practical Training

No significant changes were observed in the ER of men after practical training (*p* = 0.261) (Table 2). Regarding women, results showed a significant change in ER after the practical training period (*p* < 0.001) (Table 2). The analysis of the differences adjusted by Bonferroni correction revealed an increase in the proportion of women with adequate ER (41.12%; *n* = 44) (*p* < 0.001; 95% CI: 28.92–51.49) and excellent ER (29.80%; *n* = 17) (*p* < 0.001; 95% CI: 17.15–41.95) after the practical training. According to the area of practical training, a significant increase in the proportion of women with adequate ER (48.50%; *n* = 16) (*p* < 0.001; 95% CI: 25.96–64.27) and excellent ER (37.50%; *n* = 6) (*p* < 0.001; 95% CI: 8.49–59.64) was observed in those who had undergone training in Physical Rehabilitation (Table 4). Similarly, a significant increase was observed in the proportion of women who showed adequate ER (41.46%; *n* = 17) (*p* < 0.001; 95% CI: 20.56–57.51) among those who had undergone training in the area of Geriatrics and Gerontology (Table 4). No significant changes were observed in those who had received training in the areas of Mental Health, Child Care or in non-conventional areas.

## Discussion

The relation between the practical training of OT students and changes in EI has been addressed by a number of studies, although results have been inconclusive. It should be noted that none of these studies took into account the area of practical training (Gribble et al., 2017, 2018). Consequently, the objectives were to determine whether there were changes in the self-perceived EI of OT students after exposure to their first practical training rotation and analyze whether these changes differed depending on the area in which this practical training took place, an aspect that has not yet been taken into account, to the best of the knowledge of the authors.

The findings of the present study revealed an improvement in the self-perceived EI of OT students after the first practical training period. This improvement occurred specifically in all three dimensions of self-perceived EI in women. These results are partially in keeping with those obtained in one study (Gribble et al., 2018), who applied the Emotional Quotient Inventory 2.0 (EQ-i^2.0^) to measure EI and found a significant improvement in the overall EI score after practical training. These authors also observed an improvement in self-perception, decision-making, self-realization, emotional self-awareness, independence, and objectivity scores. However, they also revealed that a low score on the stress management subscale of the EQ-i^2.0^ was maintained after the practical training. Although the instrument used in the present study to measure self-perceived EI (TMMS-24) does not include a specific stress management scale, several authors have pointed out the relation between low self-perceived EI scores (in EA, EC, and ER) and high stress levels (Austin et al., 2010). Furthermore, a high EA score (≥33 in men and ≥36 in women, corresponding to the category “Must improve attention: pays too much attention”) and a low EC score have been associated with inadequate stress coping strategies (Extremera et al., 2003; Saklofske et al., 2007). By contrast, higher scores in the EC and ER dimensions have been associated with lower perceived stress levels (Extremera et al., 2007). At the beginning of the discussion it was pointed out that, in this study, self-perceived EI had improved after practical training in women (in EA, EC, and ER). These findings may support the relation between practical training and better stress coping abilities through the improvement of EI with practical training. Some studies have shown that students with a higher EI have a lower perception of stress (Pau et al., 2007; Naidoo and Pau, 2008), which has a positive effect on the cognitive and behavioral aspects of individuals (Gupta et al., 2017). In addition, exposure to practical training can make it easier for future professionals to apply the knowledge and skills learned during their training; it can also help them develop a range of emotional skills that will help them cope successfully with the complex situations they will have to face in their daily work (Poulsen et al., 2014).

Another relevant result of this study was the improvement in EC observed after the practical training period in women, among whom the proportion showing both adequate and excellent EC increased. Higher scores in EC (and also in ER) have been associated with greater empathy, that is, a greater ability to adopt the perspective of the other person in real everyday life situations and therefore see his/her point of view (Extremera and Fernández-Berrocal, 2004). Empathy is a particularly important value for healthcare professionals as it helps them to provide humane care, a task that is much more than providing good treatment or satisfaction to patients, and facilitates the delivery of person-oriented holistic care (Ceballos Vázquez, 2010).

Regarding ER, after practical training women showed an improvement in this dimension, resulting in a significant increase in the proportion of female students with adequate or excellent ER. The positive effects of a higher ER on healthcare professionals have been described by several authors. For instance, Extremera and Fernández Berrocal pointed out that healthcare professionals with higher ER and a greater belief in their own ability to regulate and modify negative emotional states showed greater levels of job satisfaction (Extremera and Fernández-Berrocal, 2005). In the same vein, another study highlighted the importance of recognizing a negative emotional state in order to understand its impact on the thought process, generate alternative emotional states, and manage the effects of this negative emotion (Rivers et al., 2013).

Respect to the influence of the area of clinical practice on changes in self-reported EI, results showed in women a significant improvement in all EI dimensions after practical training in the areas of Physical Rehabilitation and Geriatrics and Gerontology. However, in women after practical training in Mental Health, improved EA and EC but not ER. This difference may be due to the fact that patients treated in the area of Mental Health are often more complex. By contrast, in the areas of Physical Rehabilitation and in Geriatrics and Gerontology the role of the OT professional may be easier for students to understand and perform. This is likely to facilitate reasoning and clinical decision making, leading to a greater ability to manage stress (Lewis, 2010) and a greater belief among individuals in their ability to regulate their own emotional states (Extremera and Fernández-Berrocal, 2005). As stated above, the results showed a significant increase in the proportion of women with adequate EA in the area of Mental Health. This differs from the findings of a study carried out among healthcare professionals working in Acute Mental Health hospitalization units, whose EA was found to need some improvement (Piñeiro-Fraga, 2013). This difference may be due, partly at least, to the fact that in the present study the majority of students undertook their practical training in Community Mental Health Centers rather than in Acute Hospitalization Units, where patients tend to have greater clinical severity and instability. Regarding the women who underwent their practical training in the area of Geriatrics and Gerontology, an improvement was observed in all dimensions of self-perceived EI. A study carried out in professionals from geriatric centers revealed that those with higher scores in the EC and ER dimensions of the TMMS-24 exhibited greater self-awareness, self-control, empathy, and motivation, as well as better social skills, all of which led to better quality of care and health of the population that they took care of (Liébana Presa et al., 2012).

It has been proven that EI increases over time as a consequence of the maturing process that occurs throughout the development of individuals (Zeidner et al., 2010), with some controversy over the age at which this process ends. Some authors report that it takes place until the age of 40–50 (Bar-On, 1997), while others suggest that it may extend into the age of 60–70 (Gribble et al., 2018). Although the authors cannot rule out that the improvement in EI experienced by the students may be partly due to this maturing process, it could be considered that its influence is not likely to have been very relevant, given the short time interval between the two assessments performed (between seven and 9 weeks approximately for each subject). Therefore, the authors believe that the improvement in EI found in this study is more related to exposure to practical training.

Regarding future research, it would be interesting to conduct studies that not only consider self-perceived EI but also include tests that measure EI objectively and explore the relationship between both (Zeidner and Olnick-Shemesh, 2010). Another idea would be to include the assessment of clinical tutors on the development of students' professional skills during the practical training period and the relationship between this development and the different dimensions of EI.

The results obtained in this study have important implications for both research and practical settings. They support the theoretical model of Mayer, Salovey, and Caruso (Mayer et al., 2004), according to which EI is not a personality trait but rather a skill that can be modified by an appropriate training programme. The changes observed in EI after practical training could help OT students, clinical tutors and teachers to recognize that EI is flexible; thus, it can be improved through a practical structured program in the curricula, which could encourage students to develop skills that allow them to establish an appropriate relationship with their patients. Furthermore, in the context of OT, EI has been associated with clinical reasoning and the therapeutic use of the self (Chaffey et al., 2012) lead to a decrease in the work stress of occupational therapists a more satisfactory work life for them in the future (Sarrionandia et al., 2018).

This study has several limitations. First, student selection was done using non-probabilistic convenience sampling. This may limit the extrapolation of the results, although the usefulness of this method in exploratory studies such as this one has been demonstrated (Hernández et al., 2010). Second, the fact that no significant changes were observed in men in general terms and in women in the area of Child Care and other non-conventional areas, was probably due to the small sample size of male students (although it must be pointed out that the proportion of male students participating in the study is similar to the proportion of males enrolled in OT degree studies), the small sample size of women in those practical areas and also due to the use of Bonferroni *post-hoc* test for multiple comparisons, which reduces the possibility of chance associations but enhances the credibility of research results (Martínez-González et al., 2009). This may partly explain why certain results that showed a trend toward an improvement in self-perceived EI did not reach statistical significance after the practical training. Therefore, it would be advisable to design new studies including more universities and thus a larger number of students. Third, one of the researchers responsible for the recruitment of participants was also involved in teaching duties, which may have influenced in the acceptance of certain students to be included in the study and therefore could be a source of bias.

In conclusion and in view of the above, the findings suggest that EI is flexible and can be improved through practical training, helping students develop skills that allow them to establish an appropriate relationship with their patients. In the university context, the concept of EI is related to complementary skills, which are different from academic cognitive intelligence or academic performance (Costa and Faria, 2015) and can be improved through practical training.

## Ethics Statement

The study was approved by the Ethics Committee for Scientific Research of the Healthcare Area of Talavera de la Reina (CEIC Code: 6/2017) and by the Ethics and Research Committee of the University of Malaga (CEUMA: 86-2016-H).

## Author Contributions

JT-J, BP-L, and DR-A have prepared the manuscript. In addition, JT-J, together with AS-F and DR-A have carried out the statistical analyzes. OL-M, AC-S, MR-H, PC-G, AT-G, and MR-M have collaborated in data collection and in the revision of the manuscript.

### Conflict of Interest Statement

The authors declare that the research was conducted in the absence of any commercial or financial relationships that could be construed as a potential conflict of interest.
